# Traffic Crash Characteristics in Shenzhen, China from 2014 to 2016

**DOI:** 10.3390/ijerph18031176

**Published:** 2021-01-28

**Authors:** Guofa Li, Yuan Liao, Qiangqiang Guo, Caixiong Shen, Weijian Lai

**Affiliations:** 1 Institute of Human Factors and Ergonomics, College of Mechatronics and Control Engineering, Shenzhen University, Shenzhen 518060, China; guofali@szu.edu.cn (G.L.); 1810273023@email.szu.edu.cn (C.S.); laiweijian2018@email.szu.edu.cn (W.L.); 2 Department of Space, Earth and Environment, Division of Physical Resource Theory, Chalmers University of Technology, 41296 Gothenburg, Sweden; 3 Department of Civil and Environmental Engineering, University of Washington, Seattle, WA 98195, USA; guoqq17@uw.edu

**Keywords:** police-reported crashes, traffic safety in China, motor-vehicle, human driver

## Abstract

Road traffic crashes cause fatalities and injuries of both drivers/passengers in vehicles and pedestrians outside, thus challenge public health especially in big cities in developing countries like China. Previous efforts mainly focus on a specific crash type or causation to examine the crash characteristics in China while lacking the characteristics of various crash types, factors, and the interplay between them. This study investigated the crash characteristics in Shenzhen, one of the biggest four cities in China, based on the police-reported crashes from 2014 to 2016. The descriptive characteristics were reported in detail with respect to each of the crash attributes. Based on the recorded crash locations, the land-use pattern was obtained as one of the attributes for each crash. Then, the relationship between the attributes in motor-vehicle-involved crashes was examined using the Bayesian network analysis. We revealed the distinct crash characteristics observed between the examined levels of each attribute, as well the interplay between the attributes. This study provides an insight into the crash characteristics in Shenzhen, which would help understand the driving behavior of Chinese drivers, identify the traffic safety problems, guide the research focuses on advanced driver assistance systems (ADASs) and traffic management countermeasures in China.

## 1. Introduction

Road traffic crashes are a major challenge to ensuring the public health of human beings [1]. According to the Global Status Report on Road Safety 2018 [2] from the World Health Organization, the number of deaths in road crashes is estimated to be 1.35 million per year. Usually, the number of deaths in road crashes is higher in big cities with higher population density than in smaller cities [3,4]. It is estimated that 70% of the world’s population will live in cities by 2050 [3], challenging the transportation systems. Therefore, investigating the traffic crash statistics would help identify the traffic safety problems, based on which effective solutions (e.g., personalized advanced driver assistance systems, individualized driver training programs, autonomous driving systems, traffic management countermeasures) could be proposed to enhance traffic safety, especially in the big cities in developing countries like China. In China, it has been reported that the fatality rate per 100,000 registered vehicles is 6.5 times the number in the U.S. and 13.6 times the number in Japan [5], indicating that investigating the crash characteristics is urgently needed for traffic safety enhancement.

Many previous studies have examined the crash characteristics in China in the past decade. A brief review of related studies is presented in Table 1. Among the various data sources for crash characteristics analysis, police-reported crashes are one of the most important data sources. The analysis presented in this study was also based on police-reported crashes. The summary is shown in Table 1 also shows that most of the studies only focus on a specific type of crashes (e.g., crashes between vehicles and pedestrians) and the number of studies on the traffic crash characteristics in China is still limited, especially in the big cities.

Shenzhen, as one of the four biggest cities in China, has 13,020,000 registered residents on a total area of 1997 km${}^{2}$ in 2019. The median age of the population is 31 years old. The life expectancy of people is 83 years old. The main industries include high-tech companies, financial services, modern logistics, etc. In 2015, the total length of city roads was 6447 km and the road network density was 7.16 km/km${}^{2}$ in Shenzhen [6]. The number of owned motor vehicles was 0.28 per resident on average and the number of passenger-trips was 2069 million from 15,120 buses [7]. However, the traffic problems in Shenzhen have been a major limitation of its rapid development [8]. To the best of our knowledge, the traffic crash statistics with respect to various attributes (e.g., road type, crash type, crash causation, driver information, land-use patterns) in Shenzhen have not been well-reported in the previous literature.

Therefore, to fill the research gaps in the literature, the aim of this study is to investigate the traffic crash statistics in Shenzhen, China, based on the police-reported crashes from 2014 to 2016. Specifically, we are interested in answering the research questions as follows:

(1) What are the characteristics of traffic crashes in Shenzhen, such as road type, crash type, and driver profiles?

(2) What are the land-use patterns of the occurred crashes?

(3) What is the interplay between the main attributes of motor-vehicle-involved crashes?

The main contributions of this study can be summarized as follows: (1) This study extends the analysis of traffic crash characteristics from single crash type to a more complete picture of the crashes to better identify the problems that need to be solved for traffic safety enhancement. (2) This study uses clustering techniques on open data of the points of interest in urban systems to get the land-use patterns of the study area (i.e., Shenzhen). This enriches the original crash records with land-use patterns as their spatial context, resulting in a more holistic set of crash records. (3) The interplay between the attributes in motor-vehicle-involved crashes is established by using the classical Bayesian network method. The revealed interplay contributes to a condensed picture of how different the crash attributes are associated with each other. (4) This study provides an insight into the crash characteristics in one of the four biggest cities in China. These contributions would help understand the driving behavior of Chinese drivers and guide the research focuses and traffic management countermeasures to improve traffic safety in Shenzhen.

## 2. Materials and Methods

### 2.1. Original Data Materials

This study was based on a three-year (2014–2016) dataset of police-reported traffic crashes in Shenzhen, China. A traffic crash will get into the police records in the following situations: (1) a crash happens and any of the involved drivers, passengers, or pedestrians calls the police; (2) a crash happens but the involved traffic participants don’t call the police and take a long time to negotiate without achieving an agreement, which results in severe congestion and the congestion attracts the police to handle the dispute.

The data were obtained from the Road Safety Research Platform (RSRP) in China [38]. The RSRP is managed by the Traffic Management Research Institute of the Ministry of Public Security, and its aim is to establish a resource sharing platform in the big data era to better support road safety research, technological innovation, and transformation of research findings to practical applications for road safety improvement in China. This platform shares various publicly available traffic data including the traffic crash records in Shenzhen from 2014 to 2016, the driving behavior data of older drivers, etc.

In total, 237,255 crashes were reported in the 3 years where 436,412 traffic participants were involved in these crashes. Detailed information of each crash was recorded, as shown in Table 2. The category details of each road type can be found in the Code for Transport Planning on Urban Road (GB 50220-1995) [39] and the responsibility is determined by the polices according to Article 76 of the Road Traffic Safety Law of the People’s Republic of China [40]. In general, the recorded attributes of traffic crashes can be categorized into two groups including crash-related attributes (# 1–8) and the attributes of drivers involved in the crashes (# 9–12).

### 2.2. Land-Use Patterns of Crashes

Although the textual locations of the crashes were recorded, it is inconvenient to analyze the geolocation characteristics using the original textual locations in Chinese. To better understand the geolocation characteristics of the crashes, we firstly used reverse geocoding to find the GPS coordinates of the crashes from Google Maps API. Next, in order to aggregate the GPS coordinates of crashes into discrete zones, we divided the study area into hexagonal zones with a short diagonal of 500 m for each of them. The obtained hexagonal zones of the crashes were used to reveal the details of crashes that occurred in each area. However, only knowing in which zone the crashes occur is not informative enough from the perspective of policy-making [28]. Therefore, more details about the land-use patterns (which are not included in the police records) of the crash occurrence locations are needed.

Land-use pattern refers to how the land is used to support a variety of human activities. For example, high-density land use is typical in the central city with various restaurants and low-speed urban roads where the road users are mixed. Different types of land-use generate trips that can be distinct from each other in terms of vehicle type, average speed, and the interactions between drivers and pedestrians, which in turn have the potential to cause distinct road accidents [41].

The land-use pattern of the study area in this paper is characterized by 19 types of Points of Interests (POIs) including food, hotel, shopping, life services, beauty, tourism, leisure, sports, education, culture, medical services, automobile services, finance, real estate (office buildings, residential areas, dormitories), industrial zone, governmental agencies and organizations, access (exits or entrances to highways, parking lots, etc.), natural attractions, and transportation facilities. First, the Place API of Baidu Maps was used to retrieve these POIs in each zone with a radius of 500 m centering its centroid as the searching range. A vector of 19 POI type counts was thereby created to quantify the land-use pattern of each zone. Next, Principle Component Analysis (PCA) was applied to keep 95% of the variance after the normalization of Min-Max on the 19-POI vector of all the crash zones. With the output of PCA, K-means algorithm was then used to cluster the crash zones to minimize the squared error between the empirical mean of a cluster and the vectors of crash zones in the cluster [42]. A variety of different values of K from 2 to 20 were examined to maximize the silhouette value, which is an indicator that quantifies how well the samples were appropriately clustered [43]. The K value was finally determined by balancing the number of clusters and the silhouette value. A more detailed description of the application of K-means algorithm on POI clustering can be found in Gao, Janowicz, and Couclelis (2017) [43]. Finally, the land-use pattern of each crash was obtained as one of the attributes for further analysis to examine the interplay between the attributes.

### 2.3. Interplay between the Attributes Using Bayesian Network Analysis

Besides revealing the descriptive characteristics of crash occurrence, it is also important to find out the interplay between these crash attributes because they are not always independent of each other [44]. Understanding the interplay between the attributes supports data-driven policy-making [45], e.g., how the land-use pattern and road type are associated with a variety of crash causations and therefore, how to prevent them accordingly.

Therefore, based on the enriched crash attributes with land-use pattern, this study further uses Bayesian networks to explore the traffic crashes of motor-vehicle drivers regarding the interplay between the 12 attributes including day of week, time of day, weather, crash causation, road type, gender, age, responsibility, vehicle type, injuries and deaths, crash type, and land-use cluster. To conduct the Bayesian network analysis, the values of the attributes need to be discretized. Therefore, the number of deaths plus injuries is discretized into four levels (L1: 0; L2: 1∼4; L3: 5∼9; L4: ≥10), and driver age is discretized into 13 levels by using a 5-year-interval for each level (L1: <18; L2: 18∼25; L3: 26∼30; L4: 31∼35; ... ; L11: 66∼70; L12: >70; L13: Unknown). It should be noted that 18 is the legal age of being an adult in China, therefore the L1 and L2 levels of driver age are defined as <18 and 18∼25, respectively.

Bayesian networks are a class of graphical models that have proven particularly useful in describing the associations between the variables [45,46,47]. Given the set of crash attributes $X=\;\left\{X_{1},X_{2},\ldots ,X_{n}\right\},n=12$, Bayesian networks form a concise representation of the probabilistic dependencies between them via a directed acyclic graph (DAG) [48]. The nodes in DAG represent variables in a one-to-one manner and the links between the nodes characterize the dependence between the connected variables. The Bayesian networks enable the representation of the joint probability distribution of the crash attributes in X as a product of conditional probability distributions by applying the chain rule [46,48]. The detailed theories behind the Bayesian networks can be found in Korb and Nicholson (2010) [48] and Nagarajan, Scutari, and Lèbre (2013) [46]. The objective of using the Bayesian network in this study is to explore the interplay between the main crash attributes of motor-vehicle-involved crashes.

In practice, given X, the construction of a Bayesian network becomes a learning process to determine the model structure (i.e., structure learning) and the parameters (i.e., parameter learning) separately or simultaneously. In this study, we employed a score-based learning process using bnlearn R package [49], i.e., Bayesian information criterion (BIC), with Tabu Search as the learning algorithm to simultaneously learn the model structure and the parameters [46].

The source codes and the related documents are available online [50].

## 3. Results

To characterize the crash occurrence characteristics in Shenzhen, we firstly examined the influence of the recorded attributes, and then investigated the land-use patterns of the crashes to serve as an additional attribute. Based on the recorded and derived attributes, Bayesian networks were used to explore the interplay between the attributes to present a map on their interplay.

The traffic crash characteristics between these three years were examined and the results were almost the same across the three years with no statistical significance. Therefore, we combined the crash records of all these three years for a general analysis.

### 3.1. Influence of the Crash Attributes

**Figure 1:**
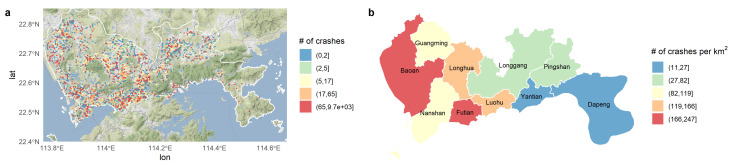
Crash distribution in Shenzhen from 2014–2016. (**a**) Number of crashes in the hexagonal zones. (**b**) Number of crashes per km${}^{2}$ by district.

The study area was divided into hexagonal cells of a 500-m short diagonal. A total of 1629 zones that have at least one crash record in each were obtained, which is shown in Figure 1. In general, crashes happened per unit area in Futian District, Baoan District, Longhua District, and Luohu District are more than the other areas because most companies and facilities (e.g., hospitals, railway stations) are in these four districts, resulting in the high travel demand in these areas. The detailed influences of the attributes on these crash records are shown as follows.

#### 3.1.1. Road Type

The crash statistics on different road types and the corresponding percentages are shown in Table 3. The results show that crashes that happened on normal urban roads overwhelm the crashes on other types of roads, accounting for 53.1% of all the crashes. The number of crashes that happened on highways ranks second, accounting for 16.5% in the three years. The numbers on other roads, city expressways, and 1st class roads account for 11.0%, 5.2%, and 3.8% of all the crashes, respectively. Considering the percentages of deaths and injuries on each road type, the results show that normal urban road/street is the most important area that needs to be studied for safety enhancement, because it is the most frequently used road type for travel in people’s frequently visited urban areas.

#### 3.1.2. Crash Type

The crash characteristics in different crash types are shown in Table 4. The crashes between vehicles have three categories, collision with motor vehicles in transport (CT3), collision with stopped vehicles (CT4), and other collision types between vehicles (CT5). In other words, any crashes that do not meet the definitions of CT3 or CT4 will be categorized as CT5. Among all the crash types, ‘collision with motor vehicles in transport’ is the dominant one, accounting for 66.5% of all the crashes, 51.0% of the deaths, 66.2% of the injuries. Among all the other crash types, pedestrian-related crashes only account for 7.0% of all the crashes (CT6, 7, and 11), but lead to 30.6% of the deaths and 15.8% of the injuries, which indicates that pedestrian safety is one of the most important research topics for traffic safety enhancement in the current autonomous driving era. The other crash type results in Table 4 show that collisions with other vehicles and fixed objects account for 7.4% and 7.6% of all the crashes, respectively.

**Figure 2:**
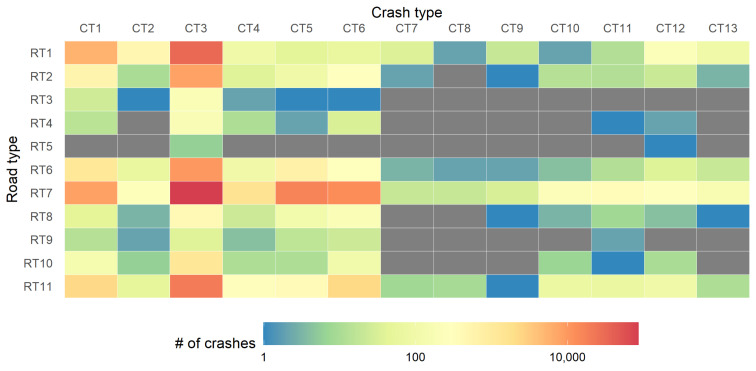
The number of crashes by crash type × road type. Unknown and others are removed.

To examine the influence of road type on the occurrence of different crash types, we illustrated the number of crashes on different road types for all the crash types in Figure 2. The Chi-square test was used to determine whether there is a statistical significance. The results show that the crash number of road types differ by crash types ($\chi^{2}=19,559,$ p<0.001). For example, more crashes between vehicles and pedestrians (CT6) happen on normal urban roads/streets (RT7). Highway (RT1) is associated with more rollover crashes (CT12) and crashes with non-fixed objects (CT2). Collisions between vehicles on a public parking lot (RT9) are not as dominant as on other road types.

#### 3.1.3. Weather

Regarding weather shown in Table 5, 83.1% of the crashes happened on sunny days, followed by light rain and cloudy accounting for 11.4% and 5.4%, respectively. A similar trend can be observed in the numbers of deaths and injuries. The recorded number of crashes in heavy rain is 54 during the three years with no deaths. The recorded crash number in haze or fog weather is 102 with 2 deaths and 29 injuries in total. It should be noted that there is no snow weather in Shenzhen because of the subtropics climate.

#### 3.1.4. Crash Causation

The descriptive crash statistics with respect to different causations are shown in Table 6. The results show that the top three causations of traffic crashes are other unsafe driver behavior (CC9), not following at a safe distance (CC8), and unsafe lane change (CC2). The percentages of crashes caused by CC9, CC8, and CC2 are 53.2%, 15.1%, and 9.8%, respectively. Among the other crash causations, it should be noted that non-motor vehicles not driving on the allowed lanes (CC16) leads to 0.7% of all the crashes, but the number of deaths caused by CC16 is 2.1%, three times the percentage of all the crashes.

**Figure 3:**
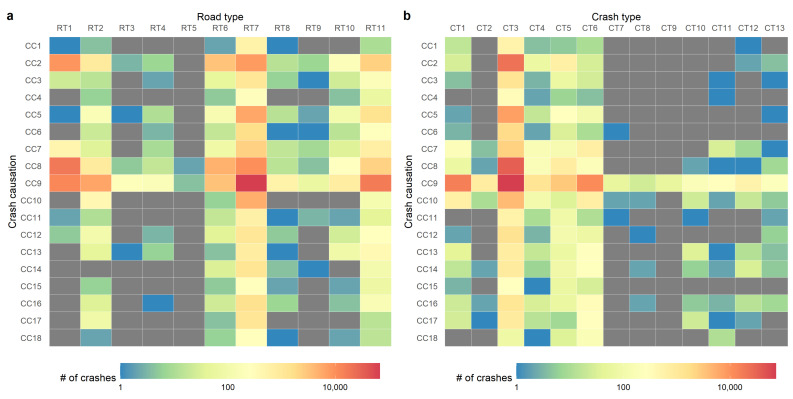
The number of crashes by (**a**) crash causation × road type, and (**b**) crash causation × crash type. Unknown and others are removed.

The number of crashes on different road types for all the crash causations are shown in Figure 3a and the information for different crash causations in the various crash types are shown in Figure 3b. The Chi-square test results show that the crash number of crash causations vary across different road types ($\chi^{2}=53,238,\;p<0.001$) and crash types ($\chi^{2}=30,304,\;p<0.001$). According to Figure 3a, a significant share of crashes on highways (RT1) are caused by unsafe lane change (CC2), not following with a safe distance (CC8), and other unsafe driver behavior (CC9). However, for urban roads such as the 1st class road (RT2), the crash causation is dominated by the other unsafe driver behavior (CC9). As for the crash type results in Figure 3b, most of the crash types are associated with other unsafe driver behavior (CC9), and this dominance is more salient for the crashes with fixed objects (CT1). For the crashes involving motor vehicles (CT3), the crash causation is more diverse with unsafe lane change (CC2), not following a safe distance (CC8), and not yielding while turning left (CC5) also frequently observed besides CC9.

#### 3.1.5. Month of Year, Day of Week, and Time of Day

**Figure 4:**
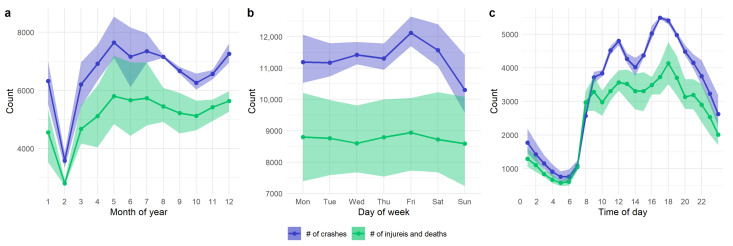
The crash occurrence with respect to time. (**a**) Month of year; (**b**) day of week; (**c**) time of day. The shadow area indicates the range from the minimum to the maximum.

The numbers of crashes from January to December are illustrated in Figure 4a. The general trends are similar across the three years. As illustrated, the number of crashes in February is the lowest point of a year because people in Shenzhen are mainly from other cities in China and they will go back their hometown for the Chinese lunar new year in February which is the most important festival in China. The numbers of crashes in January and March are also not high because people may go back their hometown earlier and come back to Shenzhen later. Similarly, the lower number in October is mainly because of the National Day holiday at the beginning of October which is one of the longest holidays in China.

**Figure 5:**
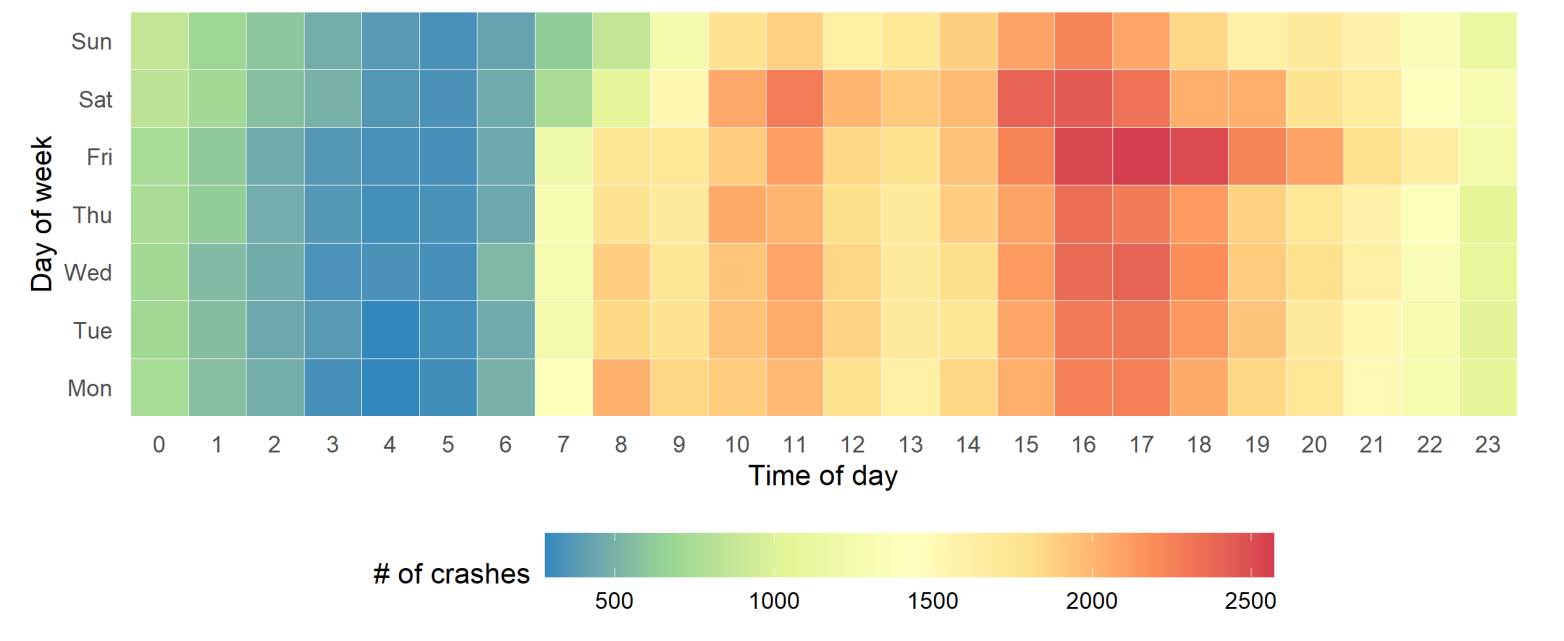
The crash occurrence patterns across time of day and day of week.

The average numbers of crashes from Monday to Sunday are shown in Figure 4b. The illustrated results show that the number of crashes is the highest on Friday and lowest on Sunday. The numbers on the other days of week are similar. As for the effect of time of day, the illustrated results in Figure 4c show that the number of crashes keeps at a high level from 9 a.m. to 9 p.m. with two peaks (i.e., 11 a.m.–1 p.m. and 4 p.m.–7 p.m.) and a trough around 2 p.m. The number continuously declines from 6 p.m. to 5 a.m., but rapidly increases from 7 a.m. to 9 a.m. To further examine the day of week and time of day patterns, the number of crashes with respect to these two attributes is illustrated in Figure 5. The results show that the crash occurrence patterns from Monday to Thursday are similar.

#### 3.1.6. Driver Gender

Among all the motor-vehicle drivers who were involved in the crashes, 69.1% of them are male and 20.9% of them are female while the rest are unknown because the polices did not record the gender information. According to the research report of metropolis’ road traffic development in China [6], the male/female proportion of the complete proportion in China is about 104:100 and the male/female proportion of all the drivers in China is about 2:1. Table 7 shows the number of crashes male and female drivers involved in with different responsibility levels. The Chi-square test shows that the driver responsibility differs between the two genders ($\chi^{2}=7888,\;p<0.001$). We define the responsibility-prone drivers as the drivers with full, major, or equal responsibility in crashes. The results in Table 7 show that the percentages of responsibility-prone drivers in the male and female groups are 53.8% and 72.8%, respectively. However, it has been frequently reported in previous studies that male drivers tend to perform more aggressively than female drivers or at least as aggressively [51,52] with higher willingness for sensation seeking and committing unsafe driving actions such as speeding [53,54]. It needs more investigation into the observed gender difference here.

#### 3.1.7. Driver Age

Table 8 shows the number of crashes that drivers from different age groups are involved in with different responsibility levels. The Chi-square test shows that the responsibility differs between the age groups ($\chi^{2}=2506,\;p<0.001$). The age distribution of drivers is generally consistent with the overall population in Shenzhen, however, the peak age of drivers shifts slightly towards the middle (31∼35 vs. 26∼30). With the increase of driver age, the percentage of responsibility-prone drivers in all the crash-involved drivers gradually declines from the highest (61.4%) for group 19∼25 to the second-lowest (39.5%) for group >70. The general trend in Table 8 demonstrates that the percentages of responsibility-prone drivers in the age groups from 18 to 45 are all higher than 10%, accounting for 77.7% in total of all the responsibility-prone drivers. The results suggest that the driving safety of old drivers will become one of the most challenging problems of driving safety in China in about 20 years.

#### 3.1.8. Vehicle Type

As for the influence of vehicle type on drivers’ crash involvement, the results are presented in Table 9. The Chi-square test suggests that the responsibility differs between the vehicle types ($\chi^{2}=23,880,\;p<0.001$). It shows that the percentages of responsibility-prone drivers for the car, bus, truck, and motorcycle groups are 49.0%, 34.7%, 10.2%, and 1.6%, respectively. Although the total number of responsibility-prone drivers in the bus and truck group is less than the number of responsibility-prone car drivers, 54.6% of the involved bus drivers and 50.9% of the involved truck drivers take full responsibility for the occurrences, while the number for car drivers is 41.7%, lower than the numbers of bus and truck drivers. The number of motorcycle drivers is 15.9%. Therefore, once a crash involves a bus or a truck, the driver of that bus or truck is more likely to take full responsibility for the crash occurrence than car drivers and motorcycle drivers.

### 3.2. Land-Use Pattern of Crashes

**Figure 6:**
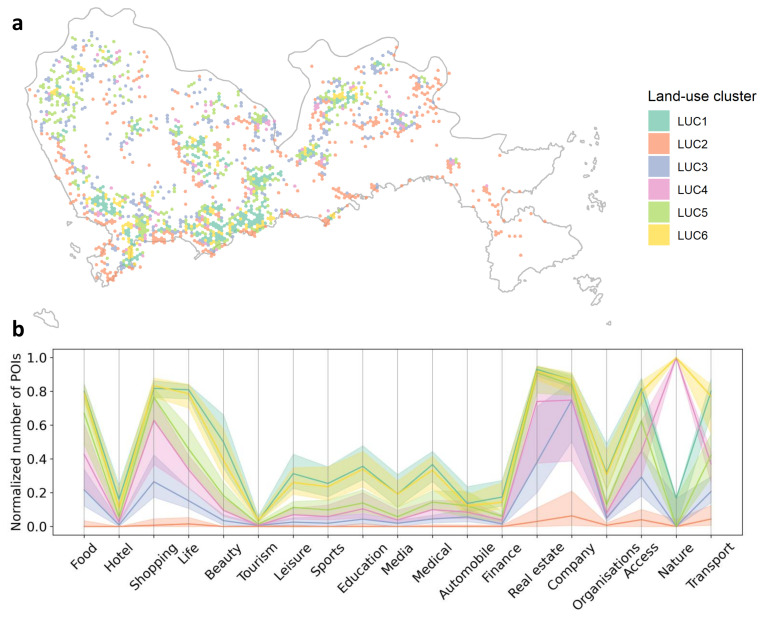
Land-use pattern results. (**a**) Land-use cluster (LUC) of the crash locations. (**b**) Normalized number of Points of Interests (POIs) for each cluster. The shadow area indicates the range from the 25th percentile value to the 75th percentile value.

In total, 6 land-use clusters were obtained based on the POI profiles. See Figure 6. The land-use clusters 4 and 5 are the first tier with the highest land-use intensity and diversity, especially for residential and commercial activities. Land-use cluster 4 is also characterized by a greater number of natural attractions than the other clusters. Clusters 1 and 6 form the second tier with a moderate level of commercial land use but high residential intensity. As the third tier, land-use clusters 2 and 3 are mainly associated with the rural and industrial areas, respectively.

The descriptions of each of the land-use clusters are summarized in Table 10. The results show that more than 50% of the crashes happened in the areas with high land-use intensity (LUC5, 23.9%) or rural characteristics (LUC2, 27.7%). The industrial areas (LUC3, 16.2%) and the areas with a medium level of land-use intensity and many residential places (LUC1, 12.9%) rank the third and fourth respectively with respect to the number of crashes. The rural areas (LUC2) have the largest number of crashes despite all types of activities (POIs) being sparse, which is different from the second-largest source LUC5 featured with high density and diversity of human activities.

The distinct land-use patterns of LUC2 and LUC5 have an equally large number of crashes, but in different traffic environments (road types). It turns out that 50% of the crashes in both land-use clusters happened on normal urban road/street (RT7), however, 33% of the crashes in LUC2 happened on highways (RT1) as compared with only 4.7% of the crashes in LUC5. On the contrary, there were 33% of the crashes in LUC5 happened on the first-class roads (RT2) and the other roads (RT11). LUC5 has many places where a variety of activities happen, therefore it tends to induce low-speed accidents due to congestion and complex driving environment. Differently, LUC2 mainly includes highways, thus the accidents happened in LUC2 are expected to be featured with high speeds.

The low-speed in LUC5 and the high-speed in LUC2 may lead to different crash occurrence characteristics. Hence, we examined the differences in deaths and injuries in these two land-use clusters. The results show that the number of deaths and the number of injuries are 0.0071 and 0.63 per crash in LUC2, respectively. The numbers are 0.0068 and 0.83 per crash in LUC5. The Kolmogorov–Smirnov test results show that there is no statistical significance on the number of deaths between LUC5 and LUC2. However, the number of injuries in LUC5 is significantly greater than the number in LUC2 (D=0.10, p<0.001). Therefore, our results show that the crashes that happened in high land-use intensity areas and rural areas have a similar level of fatality rates while crashes that happened in the high land-use intensity areas cause more injuries than the rural areas.

### 3.3. Interplay between the Attributes of Motor-Vehicle Driver Involved Crashes

To analyze the interplay between the examined crash attributes, we removed the ‘others’ and ‘unknown’ records for good descriptiveness of the obtained results. In total, 235,901 crash records with motor-vehicle-drivers were obtained to analyze the interplay between the crash attributes based on Bayesian networks.

**Figure 7:**
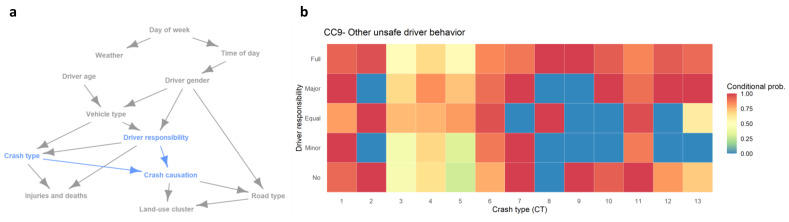
Interplay between the examined crash attributes. (**a**) Network structure with a highlighted attribute, crash causation. (**b**) Conditional probability of CC9 on crash type and the responsibility of motor-vehicle drivers.

The obtained structure of the interplay between the crash attributes is shown in Figure 7a. The results show that day of the week affects the occurrence time of the traffic crashes. The crash occurrence dependence of the time of day on the day of the week (day of week → time of day) has also been illustrated in Figure 4, where crashes tend to happen more frequently on Friday through 4 p.m. to 8 p.m. as compared with the other days of the week. The results also illustrate that female and male motor-vehicle drivers tend to involve in crashes at different times of day (time of day → driver gender). Besides, female drivers have relatively concentrated vehicle types (i.e., cars), while male drivers overwhelm the population of truck and bus drivers(driver gender → vehicle type). Moreover, driver gender also affects the taken responsibility in crashes and the road type of the occurred crashes. Driver age is also found to be associated with Vehicle type (driver age → vehicle type), and vehicle type is associated with crash type and driver responsibility (vehicle type → crash type, driver responsibility).

The illustrated results in Figure 7a also show that the number of injuries and deaths is affected by the crash type (crash type → injuries and deaths) and the role/responsibility of motor-vehicle drivers (driver responsibility → injuries and deaths). For instance, the results shown in Table 4 show sideswipe crashes with pedestrians (CT6), crushing pedestrians (CT11), and collision with stopped vehicles (CT4) tend to cause a higher number of deaths per crash as compared with the other crash types. Besides, the given results in Figure 7a also show that the responsibility of motor-vehicle drivers is associated with the crash type (driver responsibility → crash type). We can also observe that road type and crash causation are associated with the land-use pattern of the occurred crashes(crash causation → land-use cluster, road type → land-use cluster).

For detailed interpretation of the relationship between crash causation, crash type, and driver responsibility (highlighted in blue in Figure 7a), we further examined the conditional probability of crash causation CC9 (53.2% of total crashes) as dependent on the crash type and driver responsibility, as shown in Figure 7b. For the single-vehicle crash types (CT8 and CT9), almost all the crashes are caused by CC9 for the full-responsibility drivers. For the crash types that involve multiple motor-vehicles/objects (CT3-5), the crash causation is more complicated and only about half of the crashes are ascribed to CC9, different from the other crash types. For crushing pedestrian crashes (CT11), most of the crashes with drivers taking equal or no responsibility are due to CC9, while the other three responsibility groups are with lower conditional probabilities for CT11.

## 4. Discussion

### 4.1. Traffic Crash Characteristics in Shenzhen, China

Among all the crash types, ‘collision with motor vehicles in transport’ is the dominant one, accounting for 66.5% of all the crashes, 51.0% of the deaths, 66.2% of the injuries. NHTSA (2018) also reported that this crash type was the most common first harmful event in fatal crashes (39.2% of all fatal crashes) [55]. These numbers show that ‘collision with motor vehicles in transport’ challenges traffic safety the most and is urgently needed to be solved in the vehicle and transportation safety technology communities. The statistics in the U.S. show that collisions with fixed objects and non-collisions together accounted for 39.0% of all fatal crashes in 2017, and the number for rollover crashes is 17.1%. These numbers in the U.S. are much higher than the numbers in Shenzhen, China, indicating that traffic management countermeasures and intelligent systems in vehicles should be designed differently across countries.

According to the hourly records of weather in Shenzhen during 2014–2016 [56], the weather was rainy for 26.4% of the time which is greater than the percentage of crashes in rainy weather (i.e., 11.4%). However, it has been frequently reported in previous studies that drivers are more likely to be involved in crashes on rainy days mainly because of the degraded vision field [27]. This is not in contradiction with the results presented in Table 5 because people would avoid traveling on rainy days (especially in heavy rain) for safety if applicable [57]. Therefore, the reduced traveling frequency on rainy days would probably lead to a lower percentage of crashes on rainy days (11.4%) than the percentage of rainy days in the three years (26.4%).

With respect to crash causation, ‘other unsafe driver behavior while driving’ (CC9) ranks the highest, accounting for 53.2% of all the crashes and 58.5% of all the deaths in the three-year dataset of this study. This causation covers driver distraction, drowsy driving, drunk driving, driving on call, pedestrian or cyclist not following traffic rules, etc. However, the exact detailed causations were not recorded by the polices. To further improve the quality of police-reported crash records for driving safety enhancement in Shenzhen, traffic policies should clearly specify the detailed causations in the crash records in the future. Rear-end crashes have been well-reported as one of the most typical crash types, which is complied with the results in this study that ‘not following with a safe distance’ (CC8) ranks the second and accounts for 15.1% of all the crashes. Besides, frequent lane change significantly challenges driving safety. The results presented in Table 6 show ‘unsafe lane change’ (CC2) leads to 9.8% of all the crashes in the examined crash records. In general, the top three causations (CC9, CC8, and CC2) account for 78.1% of all the crashes. The statistics shown in Table 6 could provide an overview of the crash causations in Shenzhen and guide the traffic-safety-related studies, policies, and practical countermeasures to address the safety issues.

Usually, people drive to work around 8 a.m. and go back home around 4–7 p.m., resulting in congestions during morning and evening peak hours [58,59]. However, the number of crashes that occurred from 7 a.m. to 9 a.m. is far fewer than the numbers during the evening peak hours in this study. This may probably because drivers do not have many negative emotions in the morning, while their emotions would be affected by their experience during the day, resulting in negative (typically depressed and irritable) emotion states while driving in the evening peak hours [60]. Previous studies have reported that negative emotion states are closely related to aggressive driving which significantly challenges driving safety [4,61]. This explains why there are more crashes during the evening peak hours than during the morning peak hours. Differently, the number of crashes in the evening peak hours on Friday was more than the other workdays probably because of the more activities on Friday night [62]. NHTSA (2018) also reported that Friday night is the deadliest periods throughout 2017 [55]. Fewer crashes occurred on Saturday and Sunday morning, and the number of crashes on Sunday is the lowest in a week probably because people tend to enjoy their stay at home on Sunday [62]. The found crash characteristics with respect to the day of the week are consistent with the reported trends in previous studies [58,63].

Considering the percentage of responsibility-prone drivers in each age group of the crash-involved drivers, our results show that the number is the lowest for drivers younger than 18 probably because minors would only be allowed to drive under the supervision of their parents and the presence of parents degrades their aggressive driving because of the monitoring effect [64], while the number reaches the highest for age group 19∼25 mainly because of the no monitoring effect from their parents and drivers’ higher violation rates, underestimation of various violation risks, lower level of motivation to follow traffic rules, and overly involved in running red lights than older mature drivers [65]. Similarly, young male drivers involved in fatal collisions were twice as likely to be speeding as male driver from the ages of 35 to 44 in the U.S., in 2013 [66].

Compared with the drivers of passenger cars, professional drivers such as truck and bus drivers spend more time behind the wheel dealing with complicated driving tasks, and they are regulated by higher requirements on transport efficiency and fuel consumption. The higher requirements and long-time driving would lead the drivers to be fatigue and/or distracted, which would increase the probability to be involved in full responsibility crashes. Another reason leading to the higher involvement of truck and bus drivers in full responsibility crashes is that the blind zones of buses and trucks are larger than cars. Because objects (e.g., pedestrians, fixed objects on-road) in the blind zones of a vehicle are difficult to be noticed by the driver [67], the larger blind zones of buses and trucks would make the blind zone related crash risk higher than cars. This characteristic of bus/truck driving makes it attention-demanding to take care of surrounding road users and keep a safe distance from them. Therefore, ADAS systems for buses and trucks should properly address the safety challenges in the blind zones to improve driving safety, such as the blind zone warning systems.

In exploring the land-use patterns of the occurred crashes, we found that rural and central urban areas are featured with more frequent crashes than the areas with other land-use patterns. A study found that densely populated areas for public services may increase the traffic risks [68]. This is partly consistent with our finding in this study that the areas of high land-use intensity (LUC5) have the second-highest number of crashes. Interestingly, the highest number of crashes occurred in the rural areas (LUC2) with low land-use intensity in Shenzhen, different from the previous knowledge that rural areas usually have fewer or, at most, a similar number of crashes [55]. The illustrated results in Figure 6a show that the LUC2 mainly distributes along the coastline where people in Shenzhen intensively go for walking every day. Typical characteristics of the areas near the coastline in Shenzhen include that there is almost no commercial shops or stores and the around city expressways and main roads are with higher speed limits (usually 60∼80 km/h), resulting in the crash locations being clustered as rural areas. The high density of people activities and the high driving speed in LUC2 may be the leading causes of the high numbers of crashes and deaths. Our statistical analysis results also show that there is no statistical significance between LUC2 and LUC5 in Shenzhen regarding the number of fatalities, but the number of injuries in LUC5 is significantly greater than the number in LUC2. Differently, a meta-analysis on the relationship between speed and road safety [69] show that lower speed has a more positive effect on reducing fatalities in rural areas than in urban areas, but the lower speed has a more positive effect on reducing injuries in urban areas than in rural areas. According to the findings in [69], the LUC5 with lower driving speed should have fewer injuries than LUC2, which is different from our findings. This may probably because of the differences between the real rural areas in [69] and the coastline areas with rural characteristics in Shenzhen, but this needs further and deeper investigations.

In our Bayesian network analysis, we summarize the relationship between the crash attributes where the crash causation is dependent on the crash type and driver responsibility is highlighted. The results suggest that the crashes between motor vehicles and pedestrians are not always due to the misbehavior of the driver side. Even for those crashes where drivers take part of the responsibility, there are also some unsafe behaviors from the pedestrian side. Although the detailed unsafe behaviors of drivers and/or pedestrians were not recorded, one of the major unsafe driver behaviors from the existing literature is drunk driving [55]. Of the persons who were killed in crashes in 2017 in the U.S., 29% died in alcohol-impaired driving crashes [55]. This again highlights the importance of specifying and regulating the taxonomy of crash causations in police-reported crash records in Shenzhen, China. Besides, our Bayesian network analysis results show that driver age is found to be associated with vehicle type (driver age → vehicle type). This is intuitive since most bus and truck drivers in China are young or middle-aged drivers. Different vehicle types result in different patterns of crash type and driver responsibilities (vehicle type → crash type, driver responsibility), confirming the results presented in Section 3.1.8 where we found that a bus or truck driver is more likely to take full responsibility of the crash occurrence than car drivers and motorcycle drivers in bus or truck-involved crashes. Besides, road type and crash causation are also found to be associated with land-use pattern of the occurred crashes. This has been described and discussed in detail in Section 3.2 For example, LUC5 indicates the high land-use intensity areas where urban roads with low-speed-limit and crash with low-speed vehicles and pedestrians are the typical characteristics.

### 4.2. Implications of the Findings in This Study

China has an emerging driver population and cultural values that result in aberrant driving behaviors and scrambling to gain the right of way, producing a high number of crashes [5]. Although it has been frequently reported in previous studies that attributes including driver gender, age, day of the week, time of day, weather, etc. affected drivers’ involvement in crashes [52,55,70,71], few studies in the literature have investigated the crash characteristics in China from multi-aspects based on policed-reported crash records. Finding out these characteristics would help understand the behavior of Chinese road users and guide the research/application focuses to further improve road safety from the following aspects in general:

(1) Many missing values (the unknown values in the tables) or attributes (e.g., driving exposure information, property loss) and un-clarified details (e.g., the exact causation in the ‘other unsafe driver behavior’ category) can be observed in our presented results. To avoid these problems in future crash records, portable electric devices can be developed and deployed for each traffic police to record crash details with a list of required inputs including the automatically recognized GPS information, the specified unsafe driver behavior (e.g., drunk driving), etc. This would add value to the collected data for traffic characteristics analysis.

(2) Current ADAS systems have been extensively focused on the prevention of collisions with a motor vehicle in transport (CT3). However, sideswipe crashes with pedestrians (CT6) and crushing pedestrian crashes (CT11) have not been well addressed in the current literature. Our crash type results show that CT6 and CT11 crashes accounted for 6.8% and 0.2% of all the crashes but caused 19.4% and 11.1% of all the deaths, respectively. Therefore, pedestrian-related ADAS systems should be well developed to address the high death numbers in pedestrian-related crashes. Besides, the selection of typical scenes is critical for the development of ADAS systems [72,73]. The frequently observed crash causations (e.g., not following with a safe distance, unsafe lane change), crash types (e.g., CT3, CT6, CT11), occurrence locations (e.g., normal urban road/street) in this study could be selected as the typical scenes in priority for the development of solutions to improve driving safety in Shenzhen.

(3) Land-use pattern affects crash occurrence. Our results show that most of the crashes happened in rural areas (LUC2) dominated by highway collisions in Shenzhen, different from the second-ranking land-use cluster LUC5 (the areas of high land-use intensity). LUC2 is with low land-use intensity and it mainly distributes along the coastline with speed limits from 60∼80 km/h, while the LUC5 mainly distributes in the central urban areas with extensive human activities. These results indicate that the traffic management authorities in Shenzhen should design different strategies to prevent crashes in the areas with different land-use patterns. For example, more deceleration zones and alerting signals (e.g., flashing lights) can be considered in the areas where crashes frequently occur in LUC2, while more guardrails can be used to regulate pedestrian behavior and to separate motor-vehicles from pedestrians in LUC5.

(4) The Bayesian network analysis reveals the complex interplay between the examined attributes of motor-vehicle crashes, complementing the single-attribute analysis. For example, the interplay results show that vehicle type affects drivers’ responsibility in crashes (vehicle type → driver responsibility). This consolidates the finding in the single-attribute analysis that a bus or truck driver is more likely to take full responsibility for the crash occurrence than the car or motorcycle drivers if involved in crashes. This consolidated finding by the Bayesian network analysis indicates that more ADAS functions should be designed and developed to help bus and truck drivers. For instance, driver fatigue detection and warning functions for truck drivers, blind-zone monitoring and warning functions for both truck and bus drivers (as presented in detail in Section 4.1), etc.

### 4.3. Limitations and Future Work

The main limitations and future work of this study can be summarized from the following aspects. Firstly, only the crash records from 2014 to 2016 are available for analysis in this study and there are many missing values or attributes in the crash records. We will communicate with the RSRP in China and the traffic management authorities in Shenzhen to improve the quality of the collected records and use the records data of more recent years (e.g., 2018–2019) to further examine the crash characteristics in Shenzhen. Secondly, although the percentage of missing values for the crash causation attribute is low (1.1%), 53.2% of the crashes were recorded to be caused by ‘other unsafe driver behavior’ without clearly identifying the exact causations (e.g., fatigue driving, drunk driving). This would significantly limit the value of the collected causation information. We suggest the traffic management authorities in Shenzhen to detail the ‘other unsafe driver behavior’ for better development of the countermeasures to regulate or interfere drivers’ unsafe behavior. Thirdly, as reported in previous studies, the number of crashes involving electric bicycles is increasing in recent years in China [74]. Therefore, the electric bicycle involved crashes should also be recorded in much detail in the future, and the temporal variation characteristics in the crash attributes across years should be examined. Fourthly, we demonstrate the usefulness of the Bayesian network method to explore the interplay between different crash attributes in this study, however, we did not compare it with the other alternative methods. Future studies should compare the pros and cons of different methods to identify the best procedure for more insights. Fifthly, the categories of the number of deaths plus injuries are chosen according to the self-designed rules, i.e., no deaths, less than 5, less than 10, and more than 10. It would be better to conduct a sensitivity analysis to examine how the definitions of the categories affect the presented results. Last but not least, although police record is an effective way to collect traffic crash data, the possibility of under-reporting (especially severe crashes) may diminish its reliability [18]. A comparison of the fatalities data from the Chinese Center for Disease Control and Prevention and the police reports showed that the number of fatalities was about twice the police-reported number [75]. Therefore, future work should also focus on the fusion of police-reported data and other data sources (e.g., emergency medical centers, forensic institutions) to obtain more comprehensive results.

## 5. Conclusions

This study investigates the crash characteristics in Shenzhen, China and examines the interplay between the crash attributes based on the police-reported crashes from 2014 to 2016. The descriptive analysis on the number of crashes, deaths, and injuries with respect to different attributes were examined. Based on the crash records together with the derived land-use pattern of the crashes, Bayesian network clustering techniques were used to investigate the interplay between the examined attributes. The results show that higher percentages of crashes were observed on/in normal urban road/street (RT7), collision with motor vehicles in transport (CT3), sunny weather, other unsafe driver behavior (CC9) and not following with a safe distance (CC8), and Friday evening peak hours. Higher percentages of full responsibility were observed in female drivers, young adult drivers, and truck/bus drivers. The land-use pattern results show that rural areas (LUC2) and high land-use intensity areas (LUC5) were related to a higher number of crashes, deaths, and injuries. In general, the obtained interplay map from the Bayesian network analysis is complex and further investigation is needed for a detailed deeper understanding of the interplay between the examined attributes. This study provides an insight into the crash characteristics in one of the four biggest cities in China. These contributions would help understand the driving behavior of Chinese drivers and identify traffic problems to guide the research focuses and traffic management countermeasures for further improvement of traffic safety in Shenzhen, China.

## Figures and Tables

**Table 1:** A brief review of the studies on traffic crashes in China. Data source: a—yearbook; b—police-reported; c—self-collected; d—emergency medical center; and e—forensic institution.

Reference	Region in China	Area (km${}^{2}$)	Registered Residents (million)	Data Year	Data Source	Focus
[9]	China	9,600,000	1300	1951–1999	a	general crash statistics with no-specified crash type
[10]	China	9,600,000	1300	2000–2005	a	motorcycle accidents
[11]	China	9,600,000	1300	2002–2006	b	fatality crashes with no-specified crash type
[12]	China	9,600,000	1300	1951–2008	a	all reported crashes
[13]	China	9,600,000	1300	2007–2013	b	severe crashes with high numbers of fatalities and injuries
[14]	China	9,600,000	1300	2009–2013	b	crashes with more than 10 deaths with no-specified crash type
[15,16]	Guangdong	179,700	115.21	2006–2010	b	hit-and-run crashes; pedestrian-motor vehicle crashes
[17]	Xi’an	10,752	10.2	2006–2011	b	taxi crashes
[18]	Hunan	211,800	69.18	2014	b	motorcyclist crashes
[19]	Jiangxi and Shaanxi	372,500	85.42	2003–2014	b	fatigue crashes involving truck drivers
[20]	Jiangxi and Shaanxi	372,500	85.42	2006–2015	b	truck crashes on mountainous expressways
[21]	Beijing, Shanxi and Chongqing	255,512	90.06	2006–2011	b	crashes between passenger vehicles and pedestrians
[22,23]	Beijing	16,410	21.53	2009–2012	b, c	crashes between vehicles and vulnerable road users; side crashes between vehicles at intersections
[24]	Chaoyang, Beijing	470	2.585	2004	b	general crash statistics with no-specified crash type
[25]	Shanghai	6340	24.28	2008–2011	b	drunk driving crashes
[26]	downtown, Shanghai	645	-	2009	b	pedestrian crashes
[27]	Wuhan	8569	11.21	2008–2012	b	fatality crashes with no-specified crash type
[28]	downtown, Wuhan	8771 people per km${}^{2}$		2015	d	severe crashes with no-specified crash type
[29]	Taixing, Jiangsu	1172	1.18	2012–2014	b	electric bicycle crashes
[30]	Tianjin	11,966	15.61	2011–2013	e	drunk driving crashes
[31]	Changsha and Zhuzhou	23,081	12.41	2010–2016	b	truck rear-end crashes
[32]	Changsha	11,819	8.39	2014–2016	b	pedestrian crashes
[33]	Ningbo	9816	8.54	2011–2015	b	crashes between vehicles and two-wheelers
[34]	Shenzhen	1997	11.4	1994–2013	d	mortality rate of road traffic accidents
[35]	Shenzhen	1997	11.4	2010–2016	d	weather and road traffic accidents
[8]	Shenzhen	1997	11.4	2018	d	the role of road traffic injuries in public safety
[36]	Shenzhen	1997	11.4	2014	c	child safety restraint and road safety
[37]	Nanshan District, Shenzhen	187	1.3	2005–2014	b	road traffic accidents and urban planning

**Table 2:** Values of each recorded attribute.

#	Attribute	Acronym	No. of Levels	Level Description
1	Day of week	-	7	(1) Mon; (2) Tue; (3) Wed; (4) Thu; (5) Fri; (6) Sat; (7) Sun
2	Time of day	-	24	Hourly interval from 0 to 23
3	Weather	-	6	(1) Sunny; (2) Cloudy; (3) Light rain; (4) Heavy rain; (5) Haze or fog; (6) Unknown
4	Road type	RT	11	(1) Highway; (2) 1st class road; (3) 2nd class road; (4) 3rd class road; (5) 4th class road; (6) Urban expressway; (7) Urban street; (8) Residential road; (9) Public parking space; (10) Public square; (11) Other road; (12) Unknown
5	Crash causation	CC	20	(1) Motor vehicles not driving on the allowed lanes; (2) Unsafe lane change; (3) Unsafe U-turn; (4) Not following traffic rules at signalized intersections; (5) Not yielding pedestrians or straight-going vehicles while turning left; (6) Not yielding the right-hand-side vehicles at unsignalized intersections; (7) Not backing a car following traffic rules; (8) Not following with a safe distance; (9) Other unsafe driver behavior; (10) Not passing vehicles driving in the opposite direction following traffic rules; (11) Opening or closing doors to obstruct vehicles or pedestrians; (12) Driving in the opposite direction; (13) Not driving on the right-side of a road; (14) Illegal use of dedicated lanes; (15) Not following traffic signals; (16) Non-motor vehicles not driving on the allowed lanes; (17) Illegal crossing of non-motor vehicles on lanes for motor vehicles; (18) Not yielding pedestrians at the zebra-crossing area; (19) Others; (20) Unknown
6	Crash type	CT	15	(1) Collision with fixed objects; (2) Collision with non-fixed objects; (3) Collision with motor vehicles in transport; (4) Collision with stopped vehicles; (5) Other collision type between vehicles; (6) Sideswipe crashes with pedestrians; (7) Other collision type between vehicles and pedestrians; (8) Vehicle falling down cliffs; (9) Fire; (10) Passenger falling out of vehicles; (11) Crushing pedestrians; (12) Rollover; (13) Other collision type between vehicles and humans; (14) Others; (15) Unknown
7	Injuries	-	-	-
8	Deaths	-	-	-
9	Gender	-	3	(1) Female; (2) Male; (3) Unknown
10	Age	-	-	-
11	Vehicle type	-	7	(1) Car; (2) Bus; (3) Truck; (4) Motorcycle; (5) Non-motor vehicle; (6) Others; (7) Unknown
12	Responsibility	-	7	(1) Full responsibility; (2) Major responsibility; (3) Equal responsibility; (4) Minor responsibility; (5) No responsibility; (6) Unable to determine; (7) Unknown

**Table 3:** The crash characteristics on different road types.

#	Road Type	Crashes		Deaths		Injuries	
		No.	%	No.	%	No.	%
RT7	Normal urban road/street	126,047	53.1	1045	67.2	130,566	71.7
RT1	Highway	39,137	16.5	108	6.9	4293	2.4
RT6	City expressway	12,360	5.2	62	4.0	4081	2.2
RT2	1st class road	8961	3.8	67	4.3	6150	3.4
RT10	Public square	1509	0.6	11	0.7	1428	0.8
RT8	Road in residential or industrial communities	821	0.3	14	0.9	891	0.5
RT4	3rd class road	238	0.1	3	0.2	261	0.1
RT3	2nd class road	215	0.1	0	0.0	68	0.0
RT9	Public parking lot	98	0.0	5	0.3	82	0.0
RT5	4th class road	7	0.0	0	0.0	18	0.0
RT11	Other road	26,005	11.0	240	15.4	23,812	13.1
RT12	Unknown	21,857	9.2	0	0.0	10,431	5.7

**Table 4:** The crash characteristics in different crash types.

#	Crash Type	Crashes		Deaths		Injuries	
		No.	%	No.	%	No.	%
CT3	Collision with motor vehicles in transport	157,715	66.5	793	51.0	120,561	66.2
CT1	Collision with fixed objects	18,080	7.6	10	0.6	3749	2.1
CT5	Other collision type between vehicles	17,650	7.4	2	0.1	14,665	8.1
CT6	Sideswipe crashes with pedestrians	16,076	6.8	302	19.4	28,167	15.5
CT4	Collision with stopped vehicles	2103	0.9	85	5.5	1382	0.8
CT2	Collision with non-fixed objects	840	0.4	0	0.0	86	0.0
CT12	Rollover	665	0.3	1	0.1	650	0.4
CT11	Crushing pedestrians	470	0.2	173	11.1	559	0.3
CT10	Passenger falling out of vehicles	315	0.1	0	0.0	579	0.3
CT13	Other collision type between vehicles and humans	292	0.1	0	0.0	214	0.1
CT7	Other collision type between vehicles and pedestrians	67	0.0	0	0.0	30	0.0
CT9	Fire	55	0.0	0	0.0	2	0.0
CT8	Vehicle falling down cliffs	32	0.0	0	0.0	37	0.0
CT14	Others	821	0.3	181	11.6	712	0.4
CT15	Unknown	22,074	9.3	8	0.5	10,688	5.9

**Table 5:** The crash characteristics in different weather.

Weather	Crashes		Deaths		Injuries	
	No.	%	No.	%	No.	%
Sunny	197,075	83.1	1225	78.8	158,482	87.0
Cloudy	12,869	5.4	146	9.4	8022	4.4
Light rain	27,120	11.4	182	11.7	15,505	8.5
Heavy rain	54	0.0	0	0.0	23	0.0
Haze or fog	102	0.0	2	0.1	29	0.0
Unknown	35	0.0	0	0.0	20	0.0

**Table 6:** The crash characteristics with respect to different crash causations.

#	Crash Causation	Crashes		Deaths		Injuries	
		No.	%	No.	%	No.	%
CC9	Other unsafe driver behavior	126,263	53.2	909	58.5	111,140	61.0
CC8	Not following with a safe distance	35,879	15.1	45	2.9	12,733	7.0
CC2	Unsafe lane change	23,178	9.8	9	0.6	4444	2.4
CC5	Not yielding pedestrians or straight-going vehicles while turning left	7832	3.3	12	0.8	8410	4.6
CC10	Not passing vehicles driving in the opposite direction following traffic rules	5635	2.4	24	1.5	6236	3.4
CC7	Not backing a car following traffic rules	3616	1.5	27	1.7	2158	1.2
CC6	Not yielding the right-hand-side vehicles at unsignalized intersections	1930	0.8	1	0.1	1569	0.9
CC16	Non-motor vehicles not driving on the allowed lanes	1672	0.7	33	2.1	2827	1.6
CC12	Driving in the opposite direction	1538	0.6	15	1.0	2817	1.5
CC3	Unsafe U-turn	1381	0.6	4	0.3	1581	0.9
CC14	Illegal use of dedicated lanes	1288	0.5	1	0.1	2776	1.5
CC13	Not driving on the right-side of a road	1235	0.5	5	0.3	2589	1.4
CC17	Illegal crossing of non-motor vehicles on lanes for motor vehicles	1226	0.5	2	0.1	1602	0.9
CC11	Opening or closing doors to obstruct vehicles or pedestrians	694	0.3	0	0.0	1204	0.7
CC1	Motor vehicles not driving on the allowed lanes	582	0.2	0	0.0	661	0.4
CC15	Not following traffic signals	366	0.2	10	0.6	669	0.4
CC4	Not following traffic rules at signalized intersections	362	0.2	8	0.5	595	0.3
CC18	Not yielding pedestrians at the zebra-crossing area	300	0.1	17	1.1	492	0.3
CC19	Others	19,760	8.3	398	25.6	17,396	9.6
CC20	Unknown	2518	1.1	35	2.3	182	0.1

**Table 7:** The crash characteristics with respect to crash responsibility and driver gender.

Gender	Drivers	Full		Major		Equal		Minor		No		Unknown	
	%	No.	%	No.	%	No.	%	No.	%	No.	%	No.	%
Male	69.1	95,891	44.9	7451	3.5	11,743	5.5	5474	2.6	92,674	43.4	494	0.2
Female	20.9	40,982	63.4	2668	4.1	3422	5.3	566	0.9	16,930	26.2	116	0.2
Unknown	10.0	3371	11.0	205	0.7	233	0.8	26	0.1	642	2.1	26,306	85.5

**Table 8:** The crash characteristics with respect to crash responsibility and driver age. The column of Pop. shows the overall population’s age structure.

Age	Pop.	Drivers	Full		Major		Equal		Minor		No		Unknown	
	%	No.	%	No.	%	No.	%	No.	%	No.	%	No.	%	
<18	12.2	0.8	237	9.6	65	2.6	63	2.6	70	2.8	1998	81.2	27	1.1
19∼25	15.4	10.9	17,690	52.3	1351	4.0	1712	5.1	738	2.2	12,257	36.2	74	0.2
26∼30	17.4	18.4	29,256	51.5	2152	3.8	3062	5.4	1196	2.1	20,981	37.0	117	0.2
31∼35	15.5	19.6	30,149	49.8	2211	3.7	3295	5.4	1243	2.1	23,491	38.8	122	0.2
36∼40	12.0	15.7	23,652	48.9	1774	3.7	2723	5.6	1034	2.1	19,112	39.5	109	0.2
41∼45	10.2	13.1	19,568	48.2	1464	3.6	2304	5.7	916	2.3	16,231	40.0	82	0.2
46∼50	8.0	7.5	11,062	47.9	803	3.5	1319	5.7	514	2.2	9385	40.6	30	0.1
51∼55	5.0	3.0	4448	48.2	310	3.4	517	5.6	228	2.5	3706	40.1	25	0.3
56∼60	1.8	1.0	1400	43.3	102	3.2	204	6.3	73	2.3	1443	44.7	9	0.3
61∼65		0.3	435	41.1	45	4.2	65	6.1	25	2.4	489	46.2	0	0.0
66∼70	2.5	0.1	138	36.6	14	3.7	20	5.3	12	3.2	192	50.9	1	0.3
>70		0.1	137	32.6	15	3.6	14	3.3	7	1.7	246	58.6	1	0.2
Unknown	0	9.5	2072	7.1	18	0.1	100	0.3	10	0.0	715	2.4	26,319	90.0

**Table 9:** The crash characteristics with respect to crash responsibility and vehicle type.

Vehicle Type	Drivers	Full		Major		Equal		Minor		No		Unknown	
	%	No.	%	No.	%	No.	%	No.	%	No.	%	No.	%
Car	49.0	63,143	41.7	4789	3.2	7530	5.0	3023	2.0	56,186	37.1	16,715	11.0
Bus	34.7	58,575	54.6	4193	3.9	5747	5.4	1747	1.6	32,889	30.6	4195	3.9
Truck	10.2	16,035	50.9	854	2.7	1517	4.8	602	1.9	8673	27.5	3850	12.2
Motorcycle	1.6	790	15.9	397	8.0	457	9.2	600	12.1	1918	38.6	803	16.2
Others	4.5	1693	12.1	89	0.6	146	1.0	89	0.6	10,570	75.8	1353	9.7

**Table 10:** The traffic crash facts with respect to land-use cluster.

Land-Use Cluster	Description	Crashes		Deaths		Injuries	
		No.	%	No.	%	No.	%
LUC1	Medium level of land-use intensity with many residential places	30,607	12.9	178	11.4	29,361	16.1
LUC2	Rural area	65,691	27.7	469	30.2	41,058	22.5
LUC3	Industrial area	38,396	16.2	310	19.9	32,486	17.8
LUC4	High land-use intensity with many natural attractions	18,220	7.7	75	4.8	9234	5.1
LUC5	High land-use intensity	56,783	23.9	386	24.8	47,348	26.0
LUC6	Medium level of land-use intensity with many natural attractions	17,207	7.3	119	7.7	16,810	9.2
Unknown	Unknown	10,351	4.4	18	1.2	5784	3.2

## Data Availability

The data used in this study was obtained from the publicly available dataset on the Road Safety Research Platform (RSRP) in China.

## Abbreviations

The following abbreviations are used in this manuscript:

ADASs	Advanced Driver Assistance Systems
POIs	Points of Interest
PCA	Principle Component Analysis
RT	Road type
CC	Crash causation
CT	Crash type
DAG	Directed acyclic graph
BIC	Bayesian information criterion
No.	Number
NHTSA	National Highway Traffic Safety Administration
LUC	Land-use cluster
